# The Oxidative Stress and Mitochondrial Dysfunction during the Pathogenesis of Diabetic Retinopathy

**DOI:** 10.1155/2018/3420187

**Published:** 2018-09-05

**Authors:** Meng-Yu Wu, Giou-Teng Yiang, Tzu-Ting Lai, Chia-Jung Li

**Affiliations:** ^1^Department of Emergency Medicine, Taipei Tzu Chi Hospital, Buddhist Tzu Chi Medical Foundation, New Taipei 231, Taiwan; ^2^Department of Emergency Medicine, School of Medicine, Tzu Chi University, Hualien 970, Taiwan; ^3^Department of Ophthalmology, Taipei Tzu Chi Hospital, Buddhist Tzu Chi Medical Foundation, New Taipei 231, Taiwan; ^4^Research Assistant Center, Show Chwan Memorial Hospital, Changhua 500, Taiwan; ^5^Department of Medical Research, Chang Bing Show Chwan Memorial Hospital, Changhua 505, Taiwan

## Abstract

Diabetic retinopathy is one of the most serious microvascular complications induced by hyperglycemia *via* five major pathways, including polyol, hexosamine, protein kinase C, and angiotensin II pathways and the accumulation of advanced glycation end products. The hyperglycemia-induced overproduction of reactive oxygen species (ROS) induces local inflammation, mitochondrial dysfunction, microvascular dysfunction, and cell apoptosis. The accumulation of ROS, local inflammation, and cell death are tightly linked and considerably affect all phases of diabetic retinopathy pathogenesis. Furthermore, microvascular dysfunction induces ischemia and local inflammation, leading to neovascularization, macular edema, and neurodysfunction, ultimately leading to long-term blindness. Therefore, it is crucial to understand and elucidate the detailed mechanisms underlying the development of diabetic retinopathy. In this review, we summarized the existing knowledge about the pathogenesis and current strategies for the treatment of diabetic retinopathy, and we believe this systematization will help and support further research in this area.

## 1. Introduction

Diabetes mellitus (DM) is a metabolic disease characterized by hyperglycemia, due to the defects in insulin secretion and impaired insulin resistance. Diabetes, the long-term high blood sugar condition, leads to the damaging of various tissues, especially the eyes, kidneys, heart, and blood vessels, and it may aggravate other functional disorders. Diabetic retinopathy (DR) represents one of the most serious microvascular complications, in which the main pathological changes include retinal inflammation, increased vascular permeability, and abnormal angiogenesis on the surface of the retina. Previously, five classic pathways were shown to be implicated in the development of diabetic complications: polyol pathway activation, induction of the hexosamine pathway, activation of angiotensin II pathways, increase in the advanced glycosylation end product (AGE) levels in response to the activation of the cell-dependent receptors, and the activation of protein kinase C (PKC) due to the high glucose-induced peroxide overexpression [1]. Mitochondrial damage and oxidative stress are important factors affecting the development of DR [2], as they induce the production of reactive oxygen species (ROS) and the apoptosis of endothelial cells and pericytes.

Landmark clinical trials during the 1980s demonstrated that laser photocoagulation can effectively prevent the loss of vision in the patients with proliferative DR or diabetic macular edema (DME) [3, 4]. The progress of the image modalities, especially optical coherence tomography (OCT) and fluorescein angiography (FA), plays an important role in monitoring and diagnosing the disease progression and complications. Furthermore, the introduction of the intraocular administration of anti-vascular endothelial growth factor (VEGF) agents was a revolution in the management of DR, leading to the possibility of reversing the visual outcome [3–5]. Current clinical trials suggest that the anti-VEGF therapy may represent a first-line therapy for proliferative DR treatment [6]. However, although DR has been studied for a number of years *in vitro* and *in vivo*, the detailed mechanisms underlying DR pathogenesis and progression remain unclear, especially concerning the observed mitochondrial dysfunction and oxidative stress. In this review article, we summarized currently investigated mechanisms and treatments, hoping to provide a stronger foundation for the future development of targeted approaches.

## 2. Clinical Features of DR

**Figure 1:**
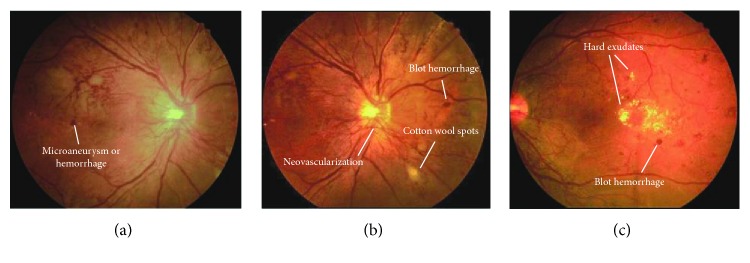
Clinical feature of diabetic retinopathy, including microaneurysm, microhemorrhage, cotton wool spots, neovascularization, and hard exudates.

Based on the fundus manifestations during DR progression, different stages of DR can be recognized: mild, moderate, severe nonproliferative, and proliferative DR [7]. In the nonproliferative DR, microaneurysms can be observed, together with some intraretinal hemorrhage and flame-shape hemorrhage, intraretinal microvascular abnormalities (IRMA), and venous caliber changes, while the proliferative DR is characterized by the presence of pathologic neovascularization (Figure 1). Proliferative DR can be further classified according to the location of the new vessels, which can be found either on the optic disc or elsewhere. Neovascularization is usually accompanied by vitreous hemorrhage, traction retinal detachment, iris neovascularization (rubeosis), and angle neovascularization with intraocular pressure elevation (neovascular glaucoma). These lesions can be found years after the diagnosis of type I DM, but they are found at the time of type II DM diagnosis [8]. An important additional category of the DR cases is diabetic macular edema (DME), which represents the most important cause of the vision loss in patients with DR. DME occurs in the DR cases with different severity of disease, even in the mild nonproliferative DR [7]. DME can be categorized into mild cases, located at the posterior pole, but distant from the center of the macula; moderate cases, where the edema is located in the macula but not the center; and severe cases, which involve the center.

## 3. Mechanisms Underlying the Development and Progression of DR

**Figure 2:**
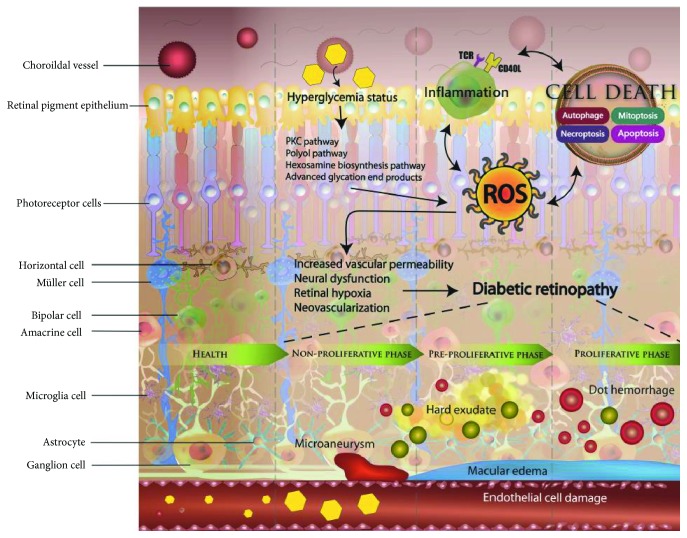
Illustration showing different mechanisms underlying diabetic retinopathy. During hyperglycemia, the excessive production of reactive oxygen species (ROS) via polyol pathway, advanced glycation end product (AGE) pathway, and protein kinase C (PKC) pathway can lead to the development of local inflammation and cell death. This vicious cycle increases vascular permeability, neural dysfunction, retinal hypoxia, and neovascularization. Neurodegeneration, inflammation, and vascular dysfunction operate in parallel and closely, which ultimately leads to the development of diabetic retinopathy.

DR is a multifactorial disease, characterized by hyperglycemia, leukostasis, microvascular damage, microinflammation, increased vascular permeability, vascular occlusion, local ischemia, and general neurodegeneration. Persistent hyperglycemia induces cellular metabolism imbalance, including excessive glucose oxidation, ROS production, local inflammation, and endothelial cell death. The detailed mechanisms underlying the accumulation of oxidative stress remain unclear. Recent studies demonstrated that five major pathways are involved in the pathogenesis of this disease, including polyol and angiotensin II pathways, AGE, PKC, and the hexosamine biosynthesis pathways [9–13]. Oxidative stress activates local inflammation and cell death. Endothelial cells on the retinal capillaries, responsible for the balancing of vascular permeability, are damaged by hyperglycemia, which further leads to fluid leakage and accumulation in the retina due to the breakdown of tight junctions between cells. Endothelial cell apoptosis, necrosis, necroptosis, and mitosis lead to local inflammation and microvascular dysfunction in the retina, which further causes blindness. The generation of ROS, inflammation, and cell death form a vicious cycle, promoting the development of DR. Furthermore, microvascular occlusions and hemorrhage activate ischemic signaling, which is followed by neovascularization through the expressions of VEGF [14, 15]. However, the neovascularized vessels are fragile, and their abnormal structure allows hemorrhaging. Progressive hemorrhage induces an increase in the VEGF expression, chronic inflammation, and retinal neurodegeneration. Neurodegeneration, inflammation, and vascular dysfunction occur in parallel and are closely interconnected (Figure 2).

Although several pathological mechanisms were reported, the details remain unclear. In this review, we focused on the increased oxidative stress, which occurs due to the polyol pathway activation leading to sorbitol accumulation, production of AGEs, activation of the PKC pathway, inflammation, and cell death.

## 4. Oxidative Stress Roles in the DR Pathogenesis

DR development is a complex pathological process. Although the mechanisms underlying this have not been completely elucidated, oxidative stress was shown to represent a key factor in this process [16]. Clinical and experimental studies demonstrated that hyperglycemia represents the primary factor leading to the pathogenesis of diabetic complications [17]. In the ischemic state, oxidative stress, superoxide dismutase, glutathione, lipid peroxide, and malondialdehyde (MDA) levels were shown to increase, while those of antioxidants decreased, thus inducing the oxidative damage of the retina [17]. *In vitro*, increased superoxide levels were observed in hyperglycemic conditions and shown to be accompanied by an increase in the hydrogen peroxide content in retinal cells [18–20]. Oxidative stress can damage cell membrane integrity as well [21], inducing apoptosis, microvascular damage, and barrier damage and ultimately leading to DR development.

### 4.1. Polyol Pathway Activation

Polyol pathway activation represents one of the processes observed under the hyperglycemia-induced oxidative stress conditions during DR pathogenesis, and this pathway is known as the sorbitol-aldose reductase pathway as well [22, 23]. Here, glucose is reduced to sorbitol and subsequently oxidized to fructose, with the help of two enzymes: aldose reductase, which converts glucose into sorbitol, and sorbitol dehydrogenase, which oxidize sorbitol into fructose [22]. Aldose reductase and sorbitol dehydrogenase require nicotinamide adenine dinucleotide phosphate (NADPH) and nicotinamide adenine dinucleotide (NAD+) to convert glucose into fructose [24]. Under hyperglycemic conditions, polyol pathway activity increases, which is followed by a decrease in the levels of NADPH that can regenerate an intracellular antioxidant, GSH [22]. The overactivation of the polyol pathway leads to the accumulation of ROS, which induces oxidative stress in cells. Under the physiological conditions, hexokinase returns to the glycolytic pathway by phosphorylating fructose into fructose-6-phosphate. However, high serum glucose levels lead to an imbalance between glycogenesis and the glycolysis pathway, favoring the accumulation of sorbitol. The imbalance in the potential energy reduction process was reported in a study examining mitochondrial dysfunction during DR pathogenesis. An excess of glucose in diabetes is converted to sorbitol by aldose reductase, but sorbitol cannot easily penetrate cellular membrane. One part of sorbitol molecules is catalyzed by the sorbitol dehydrogenase, leading to the oxidation of fructose, which is difficult to process further [25]. Therefore, sorbitol and fructose accumulate in cells, leading to an increase in osmotic pressure, edema rupture, and membrane permeability damage. Considering the effects of aldose reductase on the retina, the DR pathogenesis is induced by aldose reductase activity together with the changes in the osmotic pressure caused by the accumulation of polyhydric alcohol and the second step of the sorbitol pathway, in which SDH catalyzes the oxidation of sorbitol to fructose [26]. The reduction of NAD+ into NADH, due to hypoxia and redox imbalance, increases intracellular NADH levels, leading to cell edema, structural alterations, metabolic disorders, and microvascular lesion [27].

### 4.2. Hexosamine Pathway Activation

In the hexosamine pathway, glucose is phosphorylated and converted into fructose-6-phosphate. Glutamine provides an amino group to fructose-6-phosphate, which leads to the formation of glucosamine 6-phosphate by fructose-6-phosphate amidotransferase (GFAT) [28]. Glucosamine 6-phosphate is acetylated and isomerized to N-acetylglucosamine 6-phosphate and finally converted to diphosphate uracil-N-acetylglucosamine (UDP-GlcNAc), which can form proteoglycans, glycolipids, and glycoproteins [29]. Glucosamine can be directly phosphorylated by hexokinase as well, leading to the generation of glucosamine 6-phosphate and conversion to UDP-GlcNAc, a substrate for post-transcriptional modification of intracellular factors [30]. The hexosamine pathway was reported to mediate the toxic effects of ROS in hyperglycemia [28–31]. In the presence of increased glucose levels, a large amount of ROS is generated, which may inhibit glyceraldehyde-3-phosphate dehydrogenase (GAPDH) activity, resulting in the influx of glycolytic products to the hexosamine pathway [10, 32]. Glucosamine produced by the activated hexosamine increases H_2_O_2_ production, which further results in an increased oxidation, changes in cell endothelium, increased vascular permeability, and angiogenesis. Inhibition of GAPDH induces the AGE pathway activity as well, through the interactions with intracellular methylglyoxal, leading to the increase in retinal oxidative stress [9, 33].

### 4.3. Activation of the PKC Pathway

The PKC pathway is considered a pathway with the key role in the pathogenesis of DR. Many studies demonstrated that the activation of the PKC pathway can lead to endothelial cell damaging by increasing endothelial permeability, changing NO bioavailability, reducing prostaglandin production, inducing VEGF expression, and inducing the production of thromboxane and endothelin-1 (ET-1) [34–37]. Hyperglycemic status induces the accumulation of ROS and synthesis of diacylglycerol (DAG), leading to the PKC pathway activation. Several PKC isoforms were shown to be activated during DR pathogenesis, such as PKC-*α*, -*β*, -*δ*, and -*ε* [38, 39]. PKC-*β* activation was shown to induce the release of NO, ET-1, and VEGF in endothelial cells, leading to an increase in the retinal vascular permeability and decrease in blood flow, causing macular edema. PKC-*δ* activation induces the formation of ROS and activates the p38 and MAPK pathway, which promotes the expression of SHP-1 and NF-*κ*B, thus inhibiting the expression of platelet-derived growth factor (PDGF) and activating caspase signaling, which ultimately leads to pericyte loss and formation of microaneurysms. *In vitro*, the activity of diabetes-induced oxidative stress was shown to decrease following the administration of the PKC-*β*-specific inhibitor (LY53331), and the absence of the PKC-*β* isoform was shown to prevent ROS-mediated diabetic complications [40, 41]. Additionally, LY333531 treatment decreased PKC signaling levels, improving retinal vascular circulation [41, 42]. PKC pathway activation alters NO production through eNOS expression, directly affecting vascular tone and permeability and ultimately promoting endothelial dysfunction.

### 4.4. AGE Accumulation

Hyperglycemia leads to an increase in the nonenzymatic glycosylation of tissue macromolecules. AGEs are irreversibly cross-linked products, formed from strong glycating dicarbonyl compounds such as methylglyoxal and glyoxal [43]. The receptor for AGE (RAGE) plays an important role in the DR pathogenesis as well [44], as its activation mediates a wide range of biological effects, including ROS level increase, cytokine release, and cell function and death alterations. AGE and RAGE, accumulated in the retinal microvessels, interact directly with intracellular proteins, leading to endothelial dysfunction [45, 46]. Increased AGE accumulation induces pericyte apoptosis in the retina as well, through the activation of NF-*κ*B [47]. In the bovine retinal capillary pericytes, following the treatment with AGE solution, pericyte apoptosis and a decrease in the antioxidant activity were observed [48]. AGEs can promote the release of cytokines and VEGF, affecting vascular endothelial permeability and self-regulation and inducing inflammation.

### 4.5. Angiotensin II (ANG-II) Induces Retinal Oxidative Damage

ANG-II is the product of renin-angiotensin system (RAS) that is involved in the regulation of the systemic and local blood pressures [49]. ANG-II plays important roles in both atherosclerosis and diabetes pathogeneses [50–52]. During DR pathogenesis, this molecule induces vasoconstriction, inflammation, oxidative stress, cellular dysfunctions, angiogenesis, and fibrosis [53, 54]. Additionally, it can activate NADPH enzyme levels as well, thus increasing the production of ROS and directly damaging endothelial cells [55, 56]. Previously, ANG-II was reported to induce the production of peroxynitrite in vascular endothelial cells and to promote PARP signaling activation, which in turn activates NF-*κ*B and a release of many inflammatory cytokines, leading to the endothelial cell damage [57, 58]. These ANG-II effects can be prevented by the use of NADPH oxidase inhibitors [59, 60].

## 5. Mitochondrial Dysfunction Roles in the DR Pathogenesis

**Figure 3:**
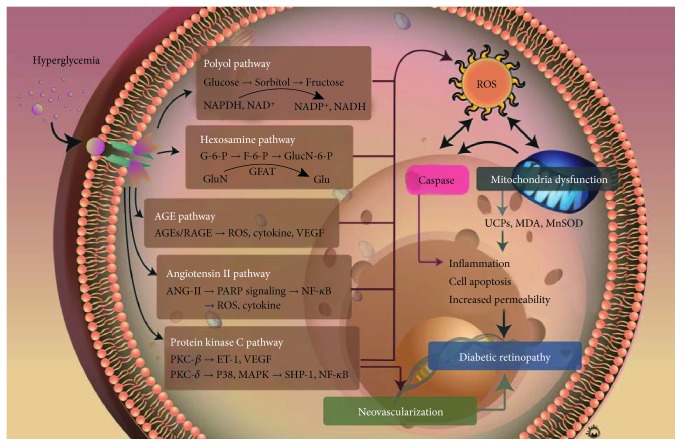
Mechanisms underlying hyperglycemia-induced oxidative stress increase that is involved in diabetic retinopathy pathogenesis.

Mitochondria are the primary source of cellular energy, involved in metabolic processes and respiration [15]. Their main role is adenosine triphosphate (ATP) production, cell metabolism control, and apoptosis regulation [61], and their dysfunction severely affects tissue homeostasis. ROS [62], superoxide dismutase, and hydroxyl radicals are mainly formed in the mitochondria. Under hyperglycemic conditions, ROS is overproduced in the retina, leading to an increase in oxidative processes and the disturbance in the mitochondrial functions, which may lead to the retinal capillary cell apoptosis [63–65]. Oxidative stress increase during hyperglycemia damages the structure and function of mitochondria [63]. The main alterations in the expression levels and activity are associated with these molecules: mitochondrial superoxide dismutase (MnSOD) [66–68], catalase (CAT) [69, 70], MDA [71–73], uncoupling proteins (UCPs) [18, 74, 75], aldose reductase, AGEs, glutathione peroxidase, nitrotyrosine (NT) [15, 76, 77], and 8-hydroxyguanosine (8-OHG and 8-OHdG) [78–80]. The UCPs, MDA, and MnSOD have been investigated the most. UCPs belong to the mitochondrial anion carrier gene family, and the uncoupling refers to the separation of ATP synthesis and mitochondrial respiration, which is achieved through proton leakage [81]. UCP functions include the reduction in the electrochemical gradient by increasing the proton leakage through the mitochondrial inner membrane, thereby reducing ROS production. Previous studies demonstrated that five isoforms of UCPs are expressed in bovine retinal microvascular endothelial cells and pericytes. UCP1, UCP2, and MnSOD were shown to be expressed in high-glucose environment [82]. Retinal neuron apoptosis in DR was observed and shown to be associated with a decrease in *MnSOD* expression [83, 84]. Mitochondrial morphology is altered in these processes as well, and their expansion can be observed in the retina of diabetic rats [20, 85]. Endothelial cells and pericytes gradually lose their original morphological features and become heterogeneous with irregular arrangement, finally leading to retinal cell apoptosis [86]. These processes induce mitochondrial ROS production, endothelial cell and pericyte apoptosis, and, ultimately, DR pathogenesis (Figure 3).

## 6. Angiogenesis and VEGF Roles during DR Pathogenesis

As early as the 1950s, scholars suggested that the DR development may be associated with retinal ischemia and hypoxia-induced neovascularization, which was first confirmed in 1994 [87, 88]. Two subtypes of VEGF exist, and VEGF2 stimulates the proliferation and migration of endothelial cells to form new blood vessels that may enable ocular microvascular leakage in the proliferative DR (PDR) patients [89]. Recent studies also found that many molecular signaling pathways associated with VEGF in patients with PDR have varying degrees of disruption, resulting in an imbalance of intravitreal angiogenesis [90–92]. The expression of placental growth factor (P1GF) in the vitreous cavity of patients with PDR is significantly increased, which further enhances VEGF signaling [90]. Additionally, the expression of connective tissue growth factor (CTGF) in the vitreous cavity of patients was shown to be significantly upregulated, and it accelerates the fibrosis process and acts in synergy with VEGF. CTGF plays an important role in the process of fibrogenesis of neovascular membrane and retinal detachment as well [91]. VEGF induces an increased expression of intracellular adhesion molecule-1 (ICAM-1), leading to the stasis and aggregation of white blood cells in the retina, gradually destroying the blood-retinal barrier and causing the damage and death of vascular endothelial cells, which ultimately leads to the formation of capillaries with no perfusion area. With the DR progression, the concentrations of VEGF and ICAM-1 in the vitreous cavity increase as well, and their levels were shown to correlate significantly [93]. VEGF also stimulates the migration of endothelial progenitor cells (EPCs) from the bone marrow to the retina and accelerates the neovascularization by inducing the release of the stem cell factor (SCF) [94, 95]. VEGF may interfere with the balance in the levels of tissue plasminogen activator (t-PA) and plasminogen activator inhibitor (PAI). The overexpression of t-PA and PAI induces extracellular matrix destruction and the degradation of the basement membrane of vascular endothelial cells [96, 97]. Increased VEGF levels may induce the expression of various inflammatory factors, such as transforming growth factor beta-1 (TGF-*β*1) and interleukin 6 (IL-6) that accelerate PDR progression [98]. Heparan sulfate is an important component of the blood-retinal barrier stroma. Abu et al. [92] demonstrated that heparanase concentrations in the vitreous cavity of the PDR patients are significantly higher compared with those in the controls, suggesting that heparanase accelerates the decomposition of heparan sulfate and the destruction of the blood-retinal barrier, together with inducing VEGF expression and promoting neovascularization. Furthermore, they demonstrated that heparan sulfate expression alterations were more severe in younger patients, which, to some extent, explains why the PDR progression is more difficult to control in younger patients with the anti-VEGF therapy [92, 99, 100].

## 7. DR Treatment Strategies

Vision loss is the most severe consequence of DR, and it can be managed using different approaches, including intraocular anti-VEGF agents and steroids for the treatment of DME, panretinal laser photocoagulation aimed at proliferative DR treatment, and surgery for vitreous hemorrhage and traction retinal detachment.

### 7.1. Laser Photocoagulation

Panretinal photocoagulation has been developed since the 1960s and represents a standard treatment for proliferative DR and DME. The principle of laser photocoagulation activity is based on the thermal effects that alleviate retinal ischemia and regulate the hemodynamics of retinal circulation. Improved retinal hypoxia and hemodynamic changes result in the regression of neovascularization and macular edema due to the reduction in the levels of VEGF and other inflammatory factors [101].

The landmark Diabetic Retinopathy Study (DRS) reported the reduction of severe vision loss in the patients with the high-risk proliferative DR and severe nonproliferative DR from 33% to 13% over 5 years following the prompt application of the panretinal photocoagulation [102]. Furthermore, the Early Treatment Diabetic Retinopathy Study (ETDRS) demonstrated that focal laser photocoagulation reduced by 50% the risk of moderate vision loss in patients with the clinically significant DME [102, 103]. Panretinal photocoagulation remains the mainstay of the proliferative DR treatment and should be especially considered for the patients with poor compliance and poor metabolic control, prior to the cataract surgery and severe cataract development, which may limit the applicability of the laser photocoagulation treatment. However, some side effects of this therapy exist, including a moderate decrease in vision, scotoma development, and secondary neovascularization development.

### 7.2. Treatment with the Intraocular Anti-VEGF Agents

In the 2000s, intraocular injection of anti-VEGF agents was introduced as a new treatment modality for DME, and this approach represents one of the most important approaches recently developed in ophthalmology [104]. Currently, three anti-VEGF treatments are used: ranibizumab (Lucentis, Genentech, South San Francisco, CA, USA), bevacizumab (Avastin, Genentech), and aflibercept (Eylea, Regeneron, Tarrytown, NY, USA).

For the treatment of DME, anti-VEGF currently represents the first-line therapy, which has replaced the focal macular laser treatment. The RESTORE study reported that the ranibizumab monotherapy or ranibizumab in combination with laser treatment demonstrated superior visual acuity gain over standard laser alone in patients with DME [105]. The results showed that the administration of ranibizumab and the deferred laser is superior to that of ranibizumab with prompt laser. For approximately 50% of the eyes treated with intravitreal ranibizumab, no further therapies were required over 5 years. These results suggest that the anti-VEGF treatment lowers the injection frequency with time, while the macular focal laser plays a less important role in the DME treatment [106, 107]. The Ranibizumab for Diabetic Macular Edema (RIDE and RISE) trials examined the effects of monthly ranibizumab injections at 0.3 mg and 0.5 mg, with the 5-year follow-up, and demonstrated that the early and regular application of ranibizumab lowers the risk of proliferative DR development. Furthermore, the use of ranibizumab was observed to prevent retinal nonperfusion [108].

Although multiple intraocular injections are required, the treatment protocol depends on the selected drug, dosage, and retreatment criteria, and different levels of visual improvement can be obtained. In the DRCR.net Protocol T and Cai and Bressler [109, 110] reports, bevacizumab, ranibizumab, and aflibercept were compared, and it was shown that all three drugs can improve vision and are well-tolerated. However, the intravitreal administration of bevacizumab was inferior to that of both aflibercept and ranibizumab. A subgroup analysis demonstrated that aflibercept has a superior effect on the vision improvement with poorer initial baseline BCVA (less than 69 letters) compared with ranibizumab and bevacizumab. The difference in the resulting visual acuity between aflibercept and ranibizumab was significant in the first year but decreased at 2 years [109, 111]. Additionally, the effects of the anti-VEGF therapy on the high-risk proliferative DR have not been clarified. Recently, the results of the DRCR.net Protocol S were presented, demonstrating that the intravitreal ranibizumab injection is not inferior to the panretinal laser photocoagulation in patients with the increased risk of proliferative DR. At 2 years, mean visual acuity improvement following the application of these two treatment modalities was shown to be similar. Area under the curve (AUC) analysis demonstrated the superiority of ranibizumab to panretinal photocoagulation, with the incidence of vitrectomy lower in the group receiving ranibizumab. Although Protocol S results confirmed only the noninferiority of ranibizumab over panretinal photocoagulation, the cost of consecutive injections was shown to be considerably lower, suggesting that the anti-VEGF therapy may represent a valuable alternative for the treatment of proliferative DR, while the combination of these therapies may be the most practical approach in the clinic [6].

More than 50% of patients with the proliferative DR were shown not to have increased VEGF levels in the vitreous fluid [88], which may explain why some proliferative DR patients are resistant to the anti-VEGF treatment. In this patient subgroup, proinflammatory cytokines most likely play pathological roles, and the application of intravitreal steroids or other cytokine inhibitors may be more plausible treatment options.

### 7.3. Intraocular Steroid Application

The molecular mechanisms involving inflammatory pathways have been proposed to underlie DME pathogenesis [112, 113]. In addition to the increase in VEGF expression, the secretion of different types of proinflammatory cytokines (TNF-*α*, IL-6, and IL-1*β*) represents a common occurrence in patients with the proliferative DR. Corticosteroids suppressing the inflammatory pathways are considered potential DME treatment options. Furthermore, inflammatory cells produce a number of angiogenic growth factors and cytokines, which can promote neovascularization [114]. Corticosteroids downregulate VEGF expression by reducing proinflammatory cytokine levels and regulating the activity of inflammatory cells. However, due to the adverse effects of corticosteroids, such as cataract development, increase in the intraocular pressure, and increased risk of endophthalmitis, and anti-VEGF therapy, they can be administered only to a selected group of patients, such as those with chronic DME.

In a number of trials, the benefits of intravitreal triamcinolone were shown to be inferior to those obtained by using laser treatment alone, during a 3-year follow-up. The DRCR.net also reported that the intravitreal triamcinolone effects lasted for less than 1 year, while the visual acuity outcome was not superior to that of the laser photocoagulation at 2 years [106].

Ozurdex (Allergan, Irvine, California, USA) is a biodegradable implant that slowly releases 0.7 mg of dexamethasone over 6 months. The Macular Edema: Assessment of Implantable Dexamethasone in Diabetes (MEAD) study evaluated the effectiveness of dexamethasone implants in patients with DME. A higher percentage of patients was shown to achieve more than 15-letter improvement over 3 years in comparison with that in the sham group (22.2% vs. 12%, *P* < 0.018) [115]. Iluvien (Alimera Sciences, Alpharetta, GA, USA), another sustained-release intravitreal implant, provides the therapeutic effects up to 36 months by slowly delivering micrograms of fluocinolone acetonide. The Fluocinolone Acetonide in Diabetic Macular Edema study evaluated the effectiveness of the low-dose (0.2 *μ*g per day) and high-dose (0.5 *μ*g per day) fluocinolone implants in patients who received at least one laser therapy, demonstrating the improvement of more than 15 letters in both fluocinolone implant-treated groups, compared with that in the control group. Furthermore, in the subgroup with chronic DME followed up for 3 years, a higher percentage of patients with Iluvien treatment showed a VA improvement of more than 15 letters than that in the control group (28.7% vs. 18.9%) [116].

### 7.4. Surgery

Vitrectomy is a standard treatment for severe proliferative DR, for patients who do not respond to the panretinal photocoagulation and anti-VEGF therapy or those with persistent vitreous hemorrhage or traction retinal detachment. The DRCR.net Protocol D study investigated the effectiveness of pars plana vitrectomy and membrane peeling in patients with DME and vitreomacular traction, reporting that approximately 40% of patients had improved visual acuity, whereas 22% of patients were shown to have poorer visual acuity outcome, with less than 10-letter gain [117]. Prolonged vitreous hemorrhage and retinal detachment involving macula are common complications of proliferative DR, and these patients should receive surgical treatment. If panretinal photocoagulation has been performed previously, the reabsorption of hemorrhage can be observed, but the surgery is indicated if vitreous hemorrhage persists for longer than 6 months [118, 119]. According to the results of the Diabetic Retinopathy Vitrectomy Study (DRVS), early vitrectomy is strongly suggested, especially in patients with type I diabetes, but no advantages were reported for the patients with type II diabetes [120].

Surgical intervention for the treatment of neovascular glaucoma is indicated in the presence of neovascularization and persistent elevation of intraocular pressure, even after the complete panretinal photocoagulation and anti-VEGF therapy. Glaucoma drainage implants (GDIs) have recently gained popularity for the treatment of neovascular glaucoma (NVG), and their success relies less on the control of intraocular inflammation and bleb failure rate [118, 119].

### 7.5. New Insights in the Pharmacological Management of DR

Several novel pharmacological therapies are currently developed to target the mechanisms underlying DR development and progression, and these are expected to change our approaches to the DR treatment. The main strategy is preventing the damage to the retinal microvasculature induced by the progression of diabetes. Experimental studies demonstrated that the PKC activity is significantly increased following the increase in blood sugar levels, which has been implicated in the pathogenesis of microvascular damage. Ruboxistaurin (LY333531), a specific inhibitor of PKC-*β*1 and -*β*2, has been shown to prevent microvascular complications and ischemia-associated neovascularization in the animal models of diabetes [40, 121]. Additional trials evaluating the effectiveness and safety of ruboxistaurin are currently being conducted [122–125]. Furthermore, the activity of somatostatin has been linked to the progression of DR, and early studies reported that the octreotide therapy in the patients with nonproliferative DR may decrease the need for the application of laser photocoagulation [126, 127]. However, these effects were not significantly partial in the patients with proliferative DR.

Systemic therapies were shown to reduce the risk of DR progression and to modulate retinal microvasculature via renin-angiotensin system (RAAS) and lipid metabolism. Fenofibrate is the third generation of fibric acid-derivative lipid-regulating drugs, with can prevent the progression of DR through multiple mechanisms, including the well-established lipid-lowering effect, the inhibition of the VEGF pathway, and the maintenance of the normal endothelial structure [128]. The Fenofibrate Intervention and Event Lowering in Diabetes (FIELD) study reported the effects of fenofibrate on the cardiovascular system and the considerable decrease in the need for laser photocoagulation treatment and severe DR progression [129]. Enalapril, an angiotensin-converting enzyme (ACE) inhibitor, was also reported to decrease the neovascularization and progression of the diabetes complications, but it remains unclear whether these beneficial effects are due to the hypotensive activity [130]. However, many drugs are still in the phases I and II of clinical trials, and they may increase the effectiveness of the DR therapy in the future.

## 8. Conclusions

The pathogenesis of DR is complex and has not been completely elucidated. However, its incidence and severity are high. Current treatment methods are mainly aimed at the treatment of the DMR and advanced-stage proliferative DR. The advantages and disadvantages of the existing single and combined therapies must be evaluated as well. Therefore, the observation that the accumulation of ROS induces neurovascular dysfunction through mitochondrial failure, inflammation, and cell death which are the major mechanisms underlying DR development represents an important conceptual advance in this field.
